# Proteome analysis for the global proteins in the jejunum tissues of enterotoxigenic *Escherichia coli* -infected piglets

**DOI:** 10.1038/srep25640

**Published:** 2016-05-09

**Authors:** Wenkai Ren, Jie Yin, Shuai Chen, Jielin Duan, Gang Liu, Tiejun Li, Nengzhang Li, Yuanyi Peng, Bie Tan, Yulong Yin

**Affiliations:** 1 Key Laboratory of Agro-ecological Processes in Subtropical Region, Institute of Subtropical Agriculture, Chinese Academy of Sciences; Hunan Provincial Engineering Research Center for Healthy Livestock and Poultry Production, Changsha, Hunan 410125, China; 2 University of the Chinese Academy of Sciences, Beijing 10008, China; 3 Chongqing Key Laboratory of Forage & Herbivorce, College of Animal Science and Technology, Southwest University, Chongqing 400716, China; 4 Hunan Collaborative Innovation Center for Utilization of Botanical Functional Ingredients; Hunan Collaborative Innovation Center of Animal Production Safety, Changsha, Hunan, 410128, China

## Abstract

Enterotoxigenic *Escherichia coli* (ETEC) is a common cause of diarrhea in humans and livestock. In this study, isobaric tags for relative and absolute quantitation (iTRAQ) combined with multidimensional liquid chromatography (LC) and MS analysis was used for screening the differentially expressed proteins in piglet jejunum after ETEC infection. Totally 1,897 proteins were identified with quantitative information in piglet jejunum. We identified 92 differentially expressed proteins in ETEC-induced diarrhea, of which 30 were up regulated and 62 down regulated. Most of the differentially expressed proteins were involved in intestinal function of binding, metabolic process, catalytic activity and immune responses. The inhibition of intestinal immune responses in the jejunum in ETEC-induced diarrhea was also validated by immunobloting and RT-PCR. Our study is the first attempt to analyze the protein profile of ETEC-infected piglets by quantitative proteomics, and our findings could provide valuable information with respect to better understanding the host response to ETEC infection.

Enterotoxigenic *Escherichia coli* (ETEC) is a common cause of childhood diarrhea in resource-limited regions and diarrhea in adult travelers to these areas^1^,^2^. ETEC is also an important cause of diarrhea in piglets^3^. ETEC produces several virulence factors, including colonization factors (adhesins), and/or the heat-labile (LT) and heat-stable (ST) toxins^4^. Colonization factors promote adherence to and colonization of the host small intestine, whereas enterotoxins stimulate the lining of the intestine and induce watery diarrhea. Briefly, LT is encoded by the *eltAB* gene and has the configuration of AB_5_^5^. LT is endocytosed after binding to host cell GM1 gangliosides in the gut and triggers constitutive cyclic adenosine monophosphate (cAMP) production^6^. The ST toxin, encoded by the *estA* gene, binds to the guanylate cyclase C receptor and stimulates overproduction of cyclic guanosine monophosphate (cGMP)^4^. Both cAMP and cGMP can induce the phosphorylation and activation of the cystic fibrosis transmembrane regulator (CFTR), leading to Cl^−^ secretion and watery diarrhea^4^. Recently, there is growing understanding about the influence of ETEC on intestinal functions, such as immune responses^7^,^8^,^9^,^10^, tight junction (TJ) function^11^,^12^,^13^,^14^, and autophagy^15^. However, most these compelling conclusions are from *in vitro studies*. Also, the global change of proteins in the jejunum after ETEC infection remains largely unknown. The knowledge of ETEC induced global alteration of proteins in the jejunum could provide better understanding in the pathogenesis of ETEC infection.

Unlike the conventional molecular biological technologies, proteomics technologies enable investigators to define the global profile of protein expression in specific physical or pathological statuses. Isobaric tags for relative and absolute quantitation (iTRAQ) combined with multidimensional liquid chromatography (LC) and MS analysis is a widely accepted and used quantitative proteomics^16^,^17^.

To investigate the global alteration of proteins in piglet jejunum after ETEC infection, the jejunum tissues from ETEC-induced diarrheal piglets, recovery piglets, and resistant piglets were profiled with iTRAQ analysis, using normal piglet jejunum as control. A total of 1,897 proteins were identified in all of the tested jejunum samples. 92 proteins were identified in ETEC-induced diarrhea, including 62 down-regulated proteins and 30 up-regulated proteins. The commonly affected function in the intestine were signal transduction mechanisms; posttranslational modification, protein turnover, chaperones; transcription; translation, ribosomal structure and biogenesis; and general function prediction. Gene ontology (GO) analysis revealed that the most affected biological functions might be the binding activity, catalytic activity, metabolic process and cellular process. Also, combined data from proteomics, immunobloting and RT-PCR, we have demonstrated that ETEC-induced diarrhea inhibited intestinal immune responses in the jejunum.

## Results

### ETEC-induced diarrhea affects protein abundance in the jejunum

Landrace ×  Yorkshire piglets were infected with ETEC W25K, a strain originally isolated from a piglet with diarrhea^18^. We observed variable responses to infection, with 49% (n =  51) of piglets developing diarrhea. The water content in the feces between diarrheal piglets and non-diarrheal piglets was also significantly different (n =  7, p <  0.01; 88.67 ±  1.4% vs. 68.40 ± 2.4%, respectively). With proteomics analysis, a total of 1,897 proteins were identified in the jejunum in the present study using Mascot version 2.3.01. Based on a 95% confidence level, cutoff values of 1.2-fold for up-regulated proteins and of 0.83-fold for down-regulated proteins were used to define a protein as being differently expressed protein in present study.

**Figure 1:**
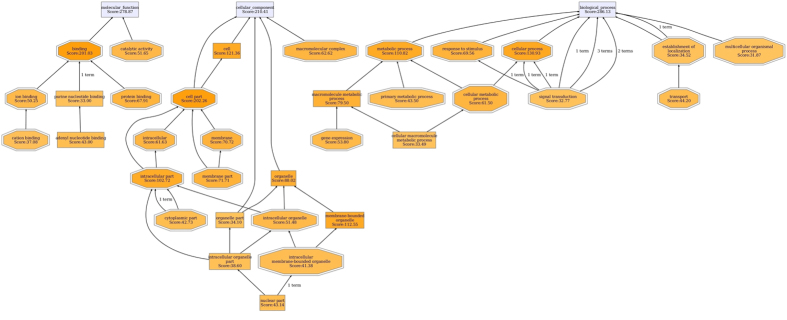
Function classification of the differentially expressed proteins between ETEC-induced diarrheal piglets and controls. ETEC-induced diarrhea in piglets were established by orally inoculation of ETEC W25K for five consecutive days at dosage of 10^10^ CFUs per day. As a control, piglets were orally infected with the same volume of LB medium. The differentially expressed proteins between 6 diarrheal piglets after ETEC infection and 6 control piglets were characterized by iTRAQ-based proteomics, and then the functional annotations of 378 differentially expressed proteins were conducted using the gene ontology (GO) annotation software. iTRAQ: isobaric tags for relative and absolute quantitation; ETEC: Enterotoxigenic *Escherichia coli*.

A total of 378 differentially expressed proteins (159 increased, 219 decreased) were found between diarrheal piglets and control piglets (Supplementary file 2). These proteins were categorized into biological processes (286), cellular components (210), and molecular functions (278) according to their annotation (Fig. 1). The most prevalent biological processes were cellular process (130), metabolic process (110), and response to the stimulus (69). The most prevalent cellular components were located in the cell part (202) and cell (121), while other proteins were assigned to the organelle (88), and macromolecular complex (62). The most predominant molecular functions were binding activity (201) and catalytic activity (51).

**Figure 2:**
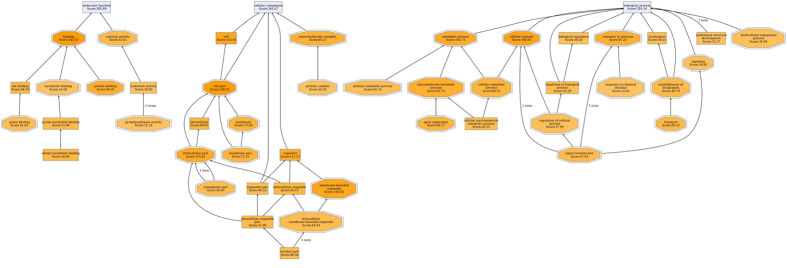
Function classification of the differentially expressed proteins between ETEC-induced diarrheal piglets and resistant piglets. ETEC-induced diarrhea in piglets were established by orally inoculation of ETEC W25K for five consecutive days at dosage of 10^10^ CFUs per day. Resistant piglets indicate the piglets that were challenged with ETEC but not suffered from diarrhea. The differentially expressed proteins between 6 diarrheal piglets after ETEC infection and 6 resistant piglets after ETEC infection were characterized by iTRAQ-based proteomics, and then the functional annotations of 450 differentially expressed proteins were conducted using the gene ontology (GO) annotation software. iTRAQ: isobaric tags for relative and absolute quantitation; ETEC: Enterotoxigenic *Escherichia coli*.

About the comparison between diarrheal piglets and resistant piglets, 450 differentially expressed proteins were found, including 186 increased proteins and 264 decreased proteins (Supplementary file 3). These differently expressed proteins were categorized into biological processes (319), cellular components (261), and molecular functions (305) according to their annotation (Fig. 2). For biological processes, the most prevalent biological processes were cellular process (160), metabolic process (110), and response to the stimulus (87). For cellular components, the most prevalent cellular components were located in the cell part (238) and cell (141). The most predominant molecular functions were binding activity (242) and catalytic activity (53).

A total of 294 differentially expressed proteins (147 increased, 147 decreased) were also found between diarrheal piglets and recovery piglets (Supplementary file 4). Meanwhile, we also found 389 differentially expressed proteins (181 increased, 208 decreased), 409 differentially expressed proteins (169 increased, 240 decreased), 435 differentially expressed proteins (242 increased, 193 decreased) between recovery piglets and control piglets (Supplementary file 5), recovery piglets and resistant piglets (Supplementary file 6), resistant piglets and control piglets (Supplementary file 7), respectively.

**Figure 3:**
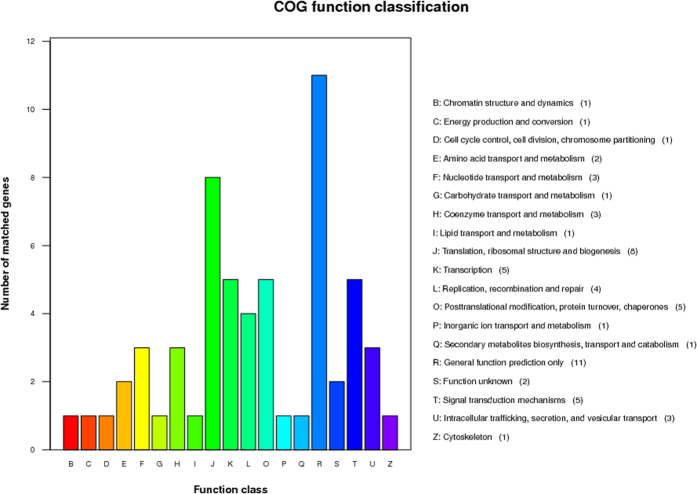
COG function classification of the differentially expressed proteins in ETEC-induced diarrheal piglets. The differentially expressed proteins among diarrheal piglets, recovery piglets, resistant piglets and control piglets were characterized by iTRAQ-based proteomics, and then the functional annotations of 92 differentially expressed proteins were conducted with COG analysis. A total of 92 differentially expressed proteins were selected based on the inclusion standard that the protein is differently expressed between diarrheal piglets and control piglets, meanwhile the level of proteins in resistant piglets or recovery piglets is closer to control piglets, compared to diarrheal piglets. iTRAQ: isobaric tags for relative and absolute quantitation; ETEC: Enterotoxigenic *Escherichia coli*.

After further inclusion standard that the protein is differently expressed between diarrheal piglets and control piglets, meanwhile the level of proteins in resistant piglets or recovery piglets is closer to control piglets, compared to diarrheal piglets, we focused our candidates down to 92 proteins, in which 62 proteins were down-regulated, while 30 proteins were up-regulated in ETEC-induced diarrhea (Supplementary file 8). These 92 differentially expressed proteins were classified into 19 groups based on their function in the intestine, i.e., general function prediction only (11); translation, ribosomal structure and biogenesis (8); transcription (5); posttranslational modification, protein turnover, chaperones (5); signal transduction mechanisms (5); nucleotide transport and metabolism (3); coenzyme metabolism (3); intracellular trafficking and secretion (3); amino acid transport and metabolism (2); function unknown (2); chromatin structure and dynamics (1); energy production and conversion (1); cell cycle control, cell division and chromosome partitioning (1); carbohydrate transport and metabolism (1); lipid transport and metabolism (1); inorganic ion transport and metabolism (1); secondary metabolite biosynthesis, transport, and catabolism (1); and cytoskeleton (1) (Fig. 3).

**Figure 4:**
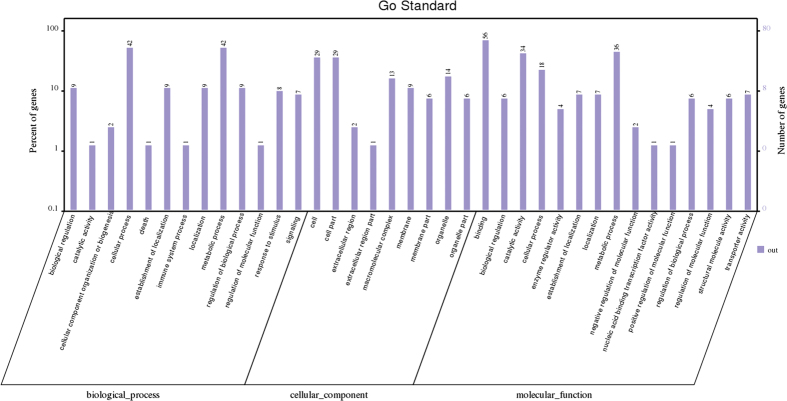
GO (gene ontology) analysis of the differentially expressed proteins in ETEC-induced diarrheal piglets. The differentially expressed proteins among diarrheal piglets, recovery piglets, resistant piglets and control piglets were characterized by iTRAQ-based proteomics, and then the functional annotations of 92 differentially expressed proteins were conducted with GO analysis. A total of 92 differentially expressed proteins were selected based on the inclusion standard that the protein is differently expressed between diarrheal piglets and control piglets, meanwhile the level of proteins in resistant piglets or recovery piglets is closer to control piglets, compared to diarrheal piglets. iTRAQ: isobaric tags for relative and absolute quantitation; ETEC: Enterotoxigenic *Escherichia coli*.

These 92 differentially expressed proteins were categorized into biological processes, cellular components, and molecular functions according to their annotation (Fig. 4). The most prevalent biological processes were cellular process (42), metabolic process (42), biological regulation (9), establishment of localization (9), localization (9) and regulation of biological process (9). The most prevalent cellular components were located in the cell (29) and cell part (29). The most predominant molecular function was binding activity (56); other major functional categories were catalytic activity (36), metabolic process (34) and cellular process (18).

**Figure 5:**
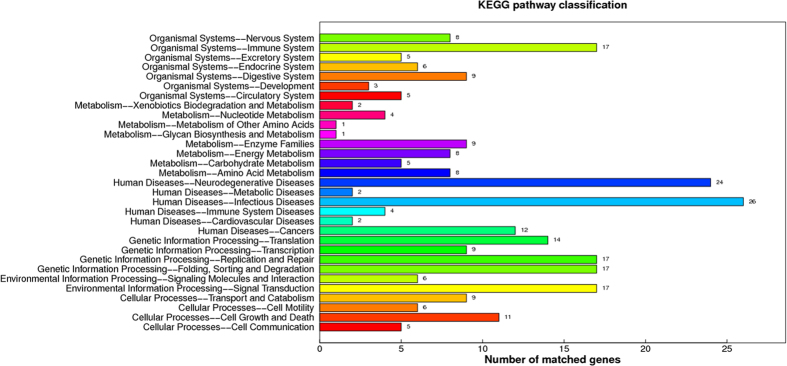
KEGG pathway analysis of the differentially expressed proteins in ETEC-induced diarrheal piglets. The differentially expressed proteins among diarrheal piglets, recovery piglets, resistant piglets and control piglets were characterized by iTRAQ-based proteomics, and then the functional annotations of 92 differently expressed proteins were conducted with KEGG pathway analysis. A total of 92 differentially expressed proteins were selected based on the inclusion standard that the protein is differentially expressed between diarrheal piglets and control piglets, meanwhile the level of proteins in resistant piglets or recovery piglets is closer to control piglets, compared to diarrheal piglets. iTRAQ: isobaric tags for relative and absolute quantitation; ETEC: Enterotoxigenic *Escherichia coli*.

Pathway analysis categorized the 92 identified proteins into 5 levels (Fig. 5). We noted that most of proteins in organismal system were associated with immune system (17); and in metabolism were enzyme families (9), energy metabolism (8) and amino acids metabolism (8). They were involved in infectious diseases (26) and neurodegenerative diseases (24) in human diseases; replication and repair (17) and folding, sorting and degradation (17) in genetic information processing; signal transduction (17) in environmental information processing; and cell growth and death (11) in cellular processes.

### ETEC-induced diarrhea inhibits the immune responses in the jejunum

**Figure 6:**
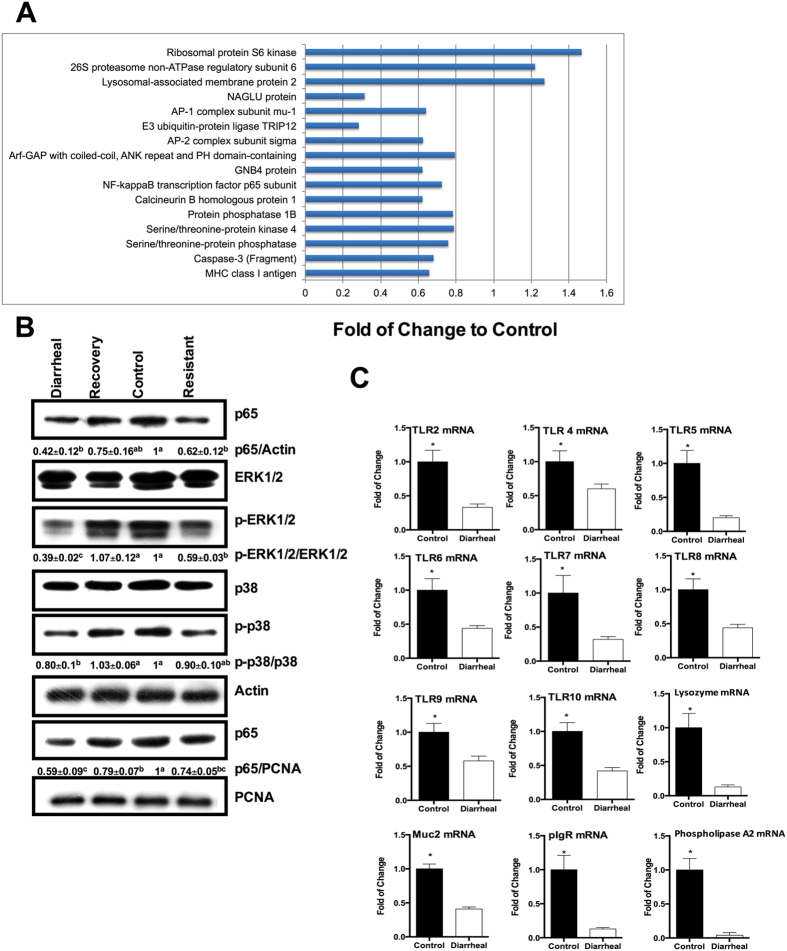
ETEC induces immune inhibition in the jejunum. (**A**) 16 differentially expressed proteins associated with immune responses were identified by proteomics. (**B**) The activation of NF-kappa B and MAPK pathway was analyzed by immunoblotting. Representative image from 5 piglets. The numbers below the image are the statistical value among different groups (n =  5; ^a–c^Means within a row with different superscripts differ, P <  0.05). (**C**) mRNA expression of innate immune genes between diarrheal piglets and control piglets (n =  6; *used to indicate a statistically significant difference, P <  0.05).

Among the 92 identified proteins, 16 proteins were associated with immune responses, and 13 of them were down-regulated in ETEC-induced diarrhea (Fig. 6A). These proteins include the NF-kappaB transcription factor p65 subunit and proteins in the mitogen-activated protein kinases (MAPK) pathway, including serine/threonine-protein kinase 4, serine/threonine-protein phosphatase, caspase-3, protein phosphatase 1B, calcineurin B homologous protein 1, and ribosomal protein S6 kinase (Fig. 6A). Thus, similar to previous evidence^9^,^10^, these results are indicating that ETEC-induced diarrhea may inhibit the NF-κ B and MAPK pathways in the jejunum. This conclusion was validated by immunoblotting, which showed that the abundance of p65 in the nucleus, as well as the abundance of p-ERK1/2 and p-p38 in the cytoplasm were decreased in diarrheal piglets, compared with the controls (Fig. 6B). Interestingly, the abundance of p65 was also lower in recovered piglets or resistant piglets compared to the control piglets (Fig. 6B). Gene expression analysis also found that diarrheal piglets had lower mRNA expression of toll-like receptor (TLR)4 and TLR5 (Fig. 6C). ETEC-induced diarrhea also reduced the mRNA expression of other TLRs on the cell surface (TLR 2, 6, 10) and endosomes (TLR 7, 8, 9) (Fig. 6C). Furthermore, ETEC-induced diarrhea significantly inhibited the mRNA expression of other indicators associated with intestinal immunity, including phospholipase A2, lysozyme, polymeric immunoglobulin receptor, and mucin 2 (Fig. 6C). Collectively, these data indicate that ETEC-induced diarrhea inhibits intestinal immune responses in the jejunum.

## Discussion

The proteomics approach has been widely applied to investigate proteome alternations in porcine infectious diseases such as porcine reproductive and respiratory syndrome (PRRS) infection^19^; porcine circovirus type 2 (PCV2) infection^20^ and porcine epidemic diarrhea virus infection^21^. In the current study, we use multidimensional LC-MS/MS coupled with iTRAQ proteomics to identify proteins that are differentially expressed in piglet jejunum during ETEC infection. Totally, 1,897 potential proteins have been detected in the jejunum tissues with 95% confidence interval based on such approach.

A total of 92 proteins have been identified in ETEC-induced diarrhea in piglet jejunum, including 62 down-regulated proteins and 30 up-regulated proteins. Of the top 10 down-regulated proteins in the jejunum, 6 proteins relate with binding function. Interestingly, one of down-regulated protein, calcineurin B homologous protein 1, plays a critical role in the function of membrane-type Na (+ )/H (+ ) exchangers (NHEs), which is essential for regulation of Na (+ ) transport in the intestinal epithelium^22^,^23^,^24^. Of the top 10 up-regulated proteins in the jejunum, 4 proteins are associated with the function of metabolism process. This is indicates that ETEC may manipulate the host metabolism for its colonization and infection. One of interested protein is asparagine synthetase, which is key enzyme for asparagine synthesis from aspartic acid. Enough content of asparagine is critical for tumor cells (leukemic cell) based on its important function in tumor cells, like inhibition of tumor cells apoptosis^25^. Thus asparaginase is developed as one of the most important chemotherapeutic agents against pediatric acute lymphoblastic leukemia. Asparaginase converts asparagine to aspartic acid in the extracellular fluid, and thus causes the depletion of extracellular asparagine for leukemic cell, resulting in the reduction of asparagine-dependent protein synthesis and ultimately leukemic cell death because most of leukemic cell putatively express low levels of asparagine synthetase or lack the ability to up-regulate the expression of asparagine synthetase when exposed to asparaginase^25^. Group A *Streptococcus* infection induces the expression of asparagine synthetase and increases the production of asparagine, which can be sensed by group A *Streptococcus* to alter the expression of genes and to increase the growth rate of group A *Streptococcus*^26^. Similarly, treatment with asparaginase lowers the level of asparagine, resulting in the inhibition of group A *Streptococcus* growth in medium and proliferation in human blood^26^. Obviously, the functions of metabolic process and binding in the jejunum tissues are most severely affected in ETEC induced diarrhea.

Using KEGG pathway analysis, we have shown that the immune responses in the jejunum are affected in ETEC induced diarrhea. Further results from immunobloting and RT-PCR suggest that ETEC-induced diarrhea inactivates NF-κ B and MAPK pathway and inhibits the intestinal expression of TLRs and other factors associated with intestinal immunity. Similarly, Wang *et al*. has demonstrated that ETEC prevents the activation of NF-κ B pathway *in vitro*^9^. Mechanically, ETEC can secrete a heat-stable proteinaceous factor, which utilizes clathrin-dependent endocytosis to enter host cells, and prevents the polyubiquitination and degradation of the NF-κ B inhibitor Iκ Bα , without affecting Iκ Bα phosphorylation^9^. However, the LT toxin from ETEC can promote the activation of NF-κ B and MAPK pathway^10^. Thus, the activation of NF-κ B and MAPK pathway in the host after ETEC infection depends on the toxins from ETEC. Indeed, in ETEC infected mouse model, the expression of factors related to intestinal innate immunity, such as the polymeric immunoglobulin receptor (pIgR), lysozyme, cryptdin-related sequence (CRS) peptides, and C-type lectins (Reg3g) are down regulated^8^. It is interesting to know which toxin mediates the inhibition of intestinal innate immunity. Also, it is widely accepted that the gut microbiota plays a pivotal role in the establishment of host-pathogen cross-talk, ultimately shaping the intestinal immune responses after infection^27^,^28^,^29^. Thus, the function of intestinal microbiota in ETEC infection and ETEC-induced diarrhea merits further investigations.

The 294 differently expressed proteins between diarrheal piglets and recovered piglets were analyzed with GO analysis. The most changed molecular functions are structural molecule activity, magnesium ion binding, cytoskeletal protein binding, identical protein binding, and actin binding. Among biological processes, protein localization, protein transport, establishment of protein localization and translation are the most of influenced biological processes. The data are indicate that these molecular functions and biological processes might be implicated in the recovery progression from ETEC induced diarrhea. Magnesium ion has been shown to effect intestinal sodium and chloride absorption, leading to affect the progression of diarrhea^30^,^31^.

Summarily, 92 differentially expressed proteins are identified in ETEC-induced diarrhea in piglet model. These proteins may be associated with intestinal function of binding, metabolic process and catalytic activity. Combined data from proteomics, immunobloting and RT-PCR, we show that ETEC-induced diarrhea inhibits intestinal immune responses in the jejunum. The identified proteins in this study with proteomics may provide useful clue in the further search in the interaction between host and ETEC.

## Materials and Methods

### Bacterial strains and antibodies

This study used the *Escherichia coli* F4-producing strain W25K (hereafter referred as ETEC; O149:K91, K88ac; LT, STb, EAST), which was originally isolated from a diarrheal piglet^18^. Antibodies against p65 (CST 6956), ERK1/2 (CST 4695), p-ERK1/2 (CST 4370), p38 (CST 8690), and p-p38 (CST 4511) were purchased from Cell Signaling Technology (Danvers, MA, USA). Antibodies against Actin (Sc-47778), and PCNA (Sc-56) were purchased from Santa Cruz Biotechnology, Inc. (Dallas, Texas, USA).

### ETEC infection of piglets

This study was conducted according to the guidelines of the Institute of Subtropical Agriculture, Chinese Academy of Sciences. All experimental protocols were approved by animal ethical committee of the Institute of Subtropical Agriculture, Chinese Academy of Sciences. Piglets ([Landrace ×  Yorkshire]; 18-days old;) were purchased from ZhengDa Co., Chongqing, China and orally inoculated for five consecutive days (day 1 to 5) with ETEC W25K at dosage of 10^10^ CFUs per day. As a control, piglets were orally infected with the same volume of LB medium. Fecal consistency was scored daily as: 0 =   normal; 1 =  soft; 2 =  runny or watery. Piglets with the development of watery diarrhea were defined as diarrheal piglets and scarified at day 2, 3 and 4 when they were alive (n =  6). Piglets in control groups were scarified at day 2, 3 and 4 (n =  6). Piglets that were recovered from diarrhea were scarified at day 6 and regarded as recovery piglets (n =  6). Piglets that were challenged with ETEC but not suffered from diarrhea were scarified at day 6 and defined as resistant piglets (n =  6). The intact jejunum samples were collected after the phosphate buffered saline (PBS; pH =  7.2–7.4) washing and stored at − 80 °C until processing. Piglets were excluded from resistant group for further study if they were negative *in vitro* villous adhesion assay^32^,^33^.

### Protein extraction and normalization

Around 400 mg of jejunum tissue was homogenized by crushing in liquid nitrogen with the aid of a mortar and pestle. Then 4 ml 10% m/v trichloroacetic acid (TCA) in acetone was added to each homogenized sample, and the mixed samples were incubated at − 20 °C for 2 h. The mixtures were then centrifuged at 20,000 *g* for 30 min at 4 °C to remove the supernatant without disturbing the pellets. The pellets were suspended in the Lysis buffer (8 M Urea, 30 mM HEPES, 1 mM PMSF, 2 mM EDTA, 10 mM DTT) and sonicated in ice for 5 min. 10 mM DTT (final concentration) was used to reduce the proteins at 56 °C for 1 h and then 55 mM IAM (final concentration) was used to alkylate in the darkroom for 1 h. The reduced and alkylated protein mixtures were precipitated by adding 4 ×  volume of chilled acetone at − 20 °C for overnight. After centrifugation at 4 °C, 20,000 *g* for 30 min, the pellet was dissolved in 0.5 M TEAB (Applied Biosystems, Milan, Italy) and sonicated in ice. After centrifuging at 30,000 *g* at 4 °C, the proteins in the supernatant were collected and an aliquot of the supernatant was taken for determination of protein concentration by Bradford method using BSA as a standard. Equivalent amounts of protein from each of six different piglets were pooled to generate one common sample for each type of tissue. Thus, we obtained 4 pooled protein samples (Diarrheal piglets; Resistant piglets, Recovery piglets and Control piglets).

### Protein digestion and peptide iTRAQ labeling

100 μ g of the pooled protein samples were digested with Trypsin Gold (Promega, Madison, WI, USA) (protein:trypsin =  30:1) at 37 °C for 24 h. After the digestion with trypsin, peptides were dried by vacuum centrifugation. The dried peptides were dissolved in 0.5 M TEAB and processed according to the manufacturer’s protocol for 8-plex iTRAQ (Applied Biosystems). Briefly, one unit of iTRAQ reagent was thawed and reconstituted in isopropanol. Peptides were then labeled individually with iTRAQ tags as follows: diarrheal piglets-113; recovery piglets-114; control piglets-115 and resistant piglets-116. Following incubation for 2 h at room temperature with the iTRAQ reagent, the labeled peptide mixtures were then pooled and dried by vacuum centrifugation.

### SCX and RP nanoLC-MS/MS Analysis of Labeled Peptide

The labeled peptides were fractionated using an HPLC system (Shimadzu, Kyoto, Japan) connected to an SCX column (Luna 5u column, 4.6 mm ×  250 mm, 5 μ m, 100 Å; Phenomenex, Torrence, CA). The labeled peptides were eluted using buffer A (10 mM KH_2_PO_4_ in an aqueous solution of 25% acetonitrile and acidified to a pH of 3.0 with H_3_PO_4_). A total of 16 fractions were collected with flow rate at 1 ml/min with buffer B (buffer A with 2 M KCl) as following: 0~30 min 100% buffer A; 31~46 min 5–30% buffer B; 46~51 min 30–50% buffer B, 51–56 min 50% buffer B; 56–61 min increasing to 100% buffer B. The fractions were desalted with a Strata X C18 column (Phenomenex) and vacuum-dried.

Then, 50 μ l of 0.1% FA was added to each dried fraction tube, and 0.1 μ l of the re-dissolved solution was spotted on the target well of an Anchor-chip plate for MALDI-TOF testing. After the MALDI-TOF (Bruker Daltonics, Germany) testing, the peptides in the tubes with few peaks were pooled, resulting in 10-pooled SCX-separated fractions. Each SCX fraction was loaded 1 times on a Nano HPLC system (Ultimate3000, Dionex) mounted with a 10 cm reversed phase C18 column (ID 75 mm, 5um particles, 300 A° aperture, 10 cm) and separated over a 65 min acetonitrile gradient from 5–80% in 0.1% FA combined with a Q Exactive mass spectrometer (Thermo Fisher Scientific, MA, USA). The data were acquired using a data-dependent data acquisition mode in which, for each cycle, the 20 most abundant multiply charged peptides (2+ to 4+ ) with an m/z between 350 and 2000 were selected for MS/MS with the 15-s dynamic exclusion setting.

### Database Analysis and protein Quantification

Proteome Discoverer 1.3 (Thermo Fisher Scientific, Waltham, MA) was used to convert the raw files to MGF format. The exported MGF files were searched by Mascot 2.3.0 (Matrix Science, Boston, MA) to simultaneously identify and quantify proteins. The database of *uniprot_pig* (5/2013, 241950 sequences) was downloaded and integrated into the Mascot search engine version 2.3.0 by its database maintenance unit. All parameters were set as follows: i) trypsin was chosen as the enzyme with one missed cleavage allowed; ii) the fixed modifications of carbamidomethylation were set as Cys; iii) iTRAQ 8-plex on the N-terminal residue, iTRAQ 8-plex on tyrosine (Y), iTRAQ 8-plex on lysine (K), Gln-Pyro-Glu (N-term Q) and oxidation on methionine (M) as the variable modification; iv) the precursor mass tolerance was set to 15 ppm for precursor ions and 20 mmu for fragment ions; v) the number of distinct peptides assigned for each protein given as “unique” and “peptides”; the coverage of each protein assigned given as “coverage”. The quantification measurement for each peptide and protein was illustrated as the ratio of diseased sample to normal control one, showing as “Diarrheal piglets/Recovery piglets, Diarrheal piglets/Control piglets; Diarrheal piglets/Resistant piglets; Recovery piglets/ Control piglets; Recovery piglets/ Resistant piglets and Resistant piglets/ Control piglets”.

The prerequisites to acquire the significant changed proteins between two groups were set up as following according to previous publication^16^. First, a qualified protein for quantitative analysis should have at least one unique peptide labeled with iTRAQ. Second, false discovery rate (FDR) <1% was adopted for all the peptides. Third, the median of unique peptides was used as analysis method for protein ratio type, correction of isotope impurities and normalization of intensity median was considered for judging the differential proteome. The proteins whose p value <  0.05 were considered the believable proteins. Finally, the proteins with 1.2-fold change (>1.20 increased or < 0.83 decreased) between two groups were considered as the differential expressed ones and the fold changes in protein abundance were defined as the median ratio of all significantly matched spectra with tag signals.

### Bioinformatics Analysis

Functional annotations of differentially expressed proteins were conducted using the gene ontology (GO) annotation software. For the pathway analysis, KOBAS was used to get protein that identified KO annotations and pathway identify. The Fisher Exactly test was used for the pathway enrichment. And the pathway with p-value <  0.05 was listed.

### RT-PCR

Total RNA was isolated from liquid nitrogen frozen and ground jejunum using TRIZOL regent (Invitrogen, USA) and then treated with DNase I (Invitrogen, USA) according to the manufacturer’s instructions. Synthesis of the first strand (cDNA) was performed with oligo (dT) 20 and Superscript II reverse transcriptase (Invitrogen, USA). Primers (Supplementary file 1) were selected according to previous references. β-actin was used as an internal control to normalize target gene transcript levels. Real-time PCR was performed according to our previous studies^34^,^35^.

### Immunoblotting

Western blot analysis was conducted according to previous studies^34^,^35^. Equal amounts of proteins obtained from jejunum cytoplasmic or nuclear fractions were separated by SDS-PAGE, transferred to PVDF membranes (Millipore, MA, USA), and blocked with 5% non-fat milk in Tris-Tween buffered saline buffer (20 mM Tris, pH 7.5, 150 mM NaCl, 0.1% Tween-20) for 3 h. Primary antibodies were incubated overnight at 4 °C and HRP-conjugated secondary antibodies were incubated for 1 h at room temperature before analysis using Alpha Imager 2200 software (Alpha Innotech Corporation, CA, USA). Signal intensity was digitally quantified and normalized to actin or proliferating cell nuclear antigen (PCNA) protein abundance.

### Statistical analyses

Data are expressed as means ±  the standard error of the mean (SEM). All statistical analyses for data were performed using SPSS 16.0 software (Chicago, IL, USA). Data between two groups were analyzed by the independent samples *t*-test, while among group analyses was by the One-Way ANOVA method. Differences of p <  0.05 are considered significant.

## Additional Information

**How to cite this article**: Ren, W. *et al.* Proteome analysis for the global proteins in the jejunum tissues of enterotoxigenic *Escherichia coli* -infected piglets. *Sci. Rep.* **6**, 25640; doi: 10.1038/srep25640 (2016).

## Supplementary Material

Supplementary Information

Supplementary File 1

Supplementary File 2

Supplementary File 3

Supplementary File 4

Supplementary File 5

Supplementary File 6

Supplementary File 7
