# Supplementary material for: Proteome analysis for the global proteins in the jejunum tissues of enterotoxigenic Escherichia coli -infected piglets
Source: Sci Rep. 2016 May 9;6:25640. doi: 10.1038/srep25640 (PMC4860632; doi:10.1038/srep25640)
Supplement: Supplementary Information [file srep25640-s1.doc]

**Proteome analysis for the global proteins in the jejunum tissues of enterotoxigenic *Escherichia coli* -infected piglets**

Wenkai Ren, Jie Yin, Shuai Chen, Jielin Duan, Gang Liu, Tiejun Li, Nengzhang Li, Yuanyi Peng, Bie Tan, Yulong Yin

**Supplementary file 1** The primers used in this study.

| Genes | Accession No. | Nucleotide sequence of primers (5′–3′) | Size (bp) | Ref |
| --- | --- | --- | --- | --- |
| β-Actin | XM_003124280.3 | F: CTGCGGCATCCACGAAACT  R: AGGGCCGTGATCTCCTTCTG | 147 |  |
| MUC20 | NM_001113440 | F: CAGCAAAGACCTCTAAGATGG  R: CAGCAGGGAGACTTGGATGG | 236 |  |
| MUC4 | NM_001206344 | AGGATGCCCAATGGCTCTACT  AAGGAGGCTGGTTCCGTTGAT | 96 |  |
| MUC13 | NM_001105293.1 | GAGACTGGCTTTAGCAACCT  AGTCTATCAAACCCTCACAC | 200 |  |
| TFRC | NM_214001.1 | CGCAAAACCAAAAAGGCTAA  TCACGAGGGGCATAATCTTC | 309 | This study |
| TLR4 | NM_001293316.1 | GCCATCGCTGCTAACATCATC  CTCATACTCAAAGATACACCATCGG | 108 |  |
| TLR5 | NM_001123202.1 | CAGCGACCAAAACAGATTGA  TGCTCACCAGACAGACAACC | 122 |  |
| TLR6 | NM_213760.1 | AACCTACTGTCATAAGCCTTCATTC GTCTACCACAAATTCACTTTCTTCAG | 95 |  |
| TLR7 | NM_001097434.1 | TCAGTCAACCGCAAGTTCTG  GATGGATCTGTAGGGGAGCA | 105 |  |
| TLR8 | NM_214187.1 | AAGACCACCACCAACTTAGCC  GACCCTCAGATTCTCATCCATCC | 105 |  |
| TLR9 | XM_005669565.1 | CACGACAGCCGAATAGCAC  GGGAACAGGGAGCAGAGC | 122 |  |
| TLR10 | NM_001030534.1 | CCTGTCCAACTGCCTCATTTG CTAAGTGTTCTAAGGATGTGTTTCTG | 106 |  |
| Mucin2 | XM_005656490.1 | CCCAGAAGGGACTGTGTATG  TTGTGTTCGCTCTTGGTCAG | 213 | This study |
| PA2 | XM_005660766.1 | GGTTTGAGGTTTGGTTTGTAGC  CTCCCTTCTTGGGCTTTATTT | 83 | This study |
| PIGR | NM_214159.1 | AACCTCACCAACTTCCCAGAG  CTAATGCCCAGACCACACTTG | 98 | This study |
| Lysozyme | NM_214392.2 | GGCGAACTGGGTGTGTTT  GGGTGTCTTGCCATCATTACA | 133 | This study |

F=forward; S=reverse.

**Supplementary file 2** Differently expressed proteins between diarrheal piglets and control piglets.

**Supplementary file 3** Differently expressed proteins between diarrheal piglets and resistant piglets.

**Supplementary file 4** Differently expressed proteins between diarrheal piglets and recovery piglets.

**Supplementary file 5** Differently expressed proteins between recovery piglets and control piglets.

**Supplementary file 6** Differently expressed proteins between recovery piglets and resistant piglets.

**Supplementary file 7** Differently expressed proteins between control piglets and resistant piglets.

**Supplementary file 8** 92 differently expressed proteins are selected in ETEC-induced diarrhea.

**References**

1. Yin, J., et al., *Birth oxidative stress and the development of an antioxidant system in newborn piglets.* Free Radic Res, 2013. **47**(12): p. 1027-35.

2. Zhou, C., et al., *Differential gene expression profiling of porcine epithelial cells infected with three enterotoxigenic Escherichia coli strains.* BMC Genomics, 2012. **13**: p. 330.

3. Sargeant, H.R., et al., *Dietary zinc oxide affects the expression of genes associated with inflammation: Transcriptome analysis in piglets challenged with ETEC K88.* Vet Immunol Immunopathol, 2010. **137**(1-2): p. 120-9.

4. Collado-Romero, M., et al., *Quantitative analysis of the immune response upon Salmonella typhimurium infection along the porcine intestinal gut.* Veterinary Research, 2010. **41**(2).
